# Randomized Controlled Trial of Etodolac versus Combination of Etodolac and Eperisone in Patients of Knee Osteoarthritis 

**DOI:** 10.1155/2013/273695

**Published:** 2013-09-30

**Authors:** Navjot Kaur, Harinder Singh, Avinash Chander Gupta

**Affiliations:** ^1^Department of Pharmacology, Government Medical College and Rajindra Hospital, Patiala, Punjab 147001, India; ^2^Department of Pharmacology, Post Graduate Institute of Medical Education and Research (PGIMER), Chandigarh 160012, India; ^3^Department of Orthopaedics, Government Medical College and Rajindra Hospital, Patiala, Punjab 147001, India

## Abstract

*Objective*. To compare the efficacy and tolerability of etodolac versus etodolac in combination with eperisone in patients of Osteoarthritis knee. *Patients and Methods*. A prospective, randomized, open label, parallel group, comparative study was conducted in 60 patients of knee OA over a period of 2 months. Thirty patients received etodolac 600 mg once daily and 30 patients received eperisone 50 mg thrice daily in addition to etodolac 600 mg once daily for 8 weeks. Efficacy assessment was done on the basis of improvement in mean scores of spontaneous pain on Visual analog scale (VAS), pain on movement, functional capacity, joint tenderness, swelling, erythema on Likert scale, and patient's overall arthritic condition on a five-point investigator scale at the end of study period as compared with the baseline scores. Assessment of tolerability was done by recording the occurrence of adverse events. Data was analyzed using Chi square test and students *t*-test. *Results*. All the enrolled patients completed the study and were compliant to the treatment regimens that they were allocated to. Both the treatment groups showed a statistically significant improvement in all the efficacy parameters at the end of 8 weeks as compared to baseline (*P* < 0.05) with no statistically significant difference between the groups. Adverse events were few and mild in nature. *Conclusion*. Combination of etodolac and eperisone is as effective as etodolac alone in patients of OA knee. Thus, it is concluded that additional use of muscle relaxant has no adjuvant value in patients of OA knee and is not recommended. The study is registered with the Clinical Trial Registry of India vide registration number CTRI/2013/03/003442.

## 1. Introduction

Osteoarthritis (OA) is the most common chronic joint disease with a prevalence of 22%–39% in India, accounting for 30% of all joint disorders [1]. Symptomatic OA, particularly of knee and hip, is the leading cause of disability between the fourth and fifth decades of life [2]. The disease is characterized by the degeneration of articular cartilage, subchondral sclerosis, cyst, and osteophyte formation. These result in joint pain and tenderness, limited movement, crepitus, effusion, and inflammation [3]. Treatment is aimed at reducing pain, maintaining mobility, and minimizing disability. Pain control is of paramount importance in order to maintain quality of life in such patients. There is, however, no single ideal pain medication for management of arthritic pain. The WHO analgesic ladder advocates a stepped approach to the use of analgesics from these groups: simple analgesics, that is, paracetamol and nonsteroidal anti-inflammatory drugs (NSAIDs), weak opioids, that is, tramadol and codeine, and strong opioids, that is, morphine, fentanyl, pethidine, and adjuvants. Adjuvant analgesics are drugs which have weak or no analgesic action when administered alone but can enhance analgesic action when coadministered with known analgesics in difficult to manage pain. They are a diverse group of drugs that includes antidepressants, anticonvulsants, skeletal muscle relaxants, and local anaesthetics.

Etodolac is a USA FDA-approved NSAID of the pyranocarboxylic acid group. It is being increasingly used in treatment of OA on account of its potent analgesic and anti-inflammatory activity. It has some degree of COX-2 selectivity over COX-1, so frequency of gastrointestinal adverse effects is less [4]. It also inhibits generation of active oxygen species and bradykinin formation in a concentration-dependent manner [5]. Etodolac has the potential advantage of not damaging articular cartilage in vivo [6] and it has a good safety profile [7]. Recommended dose is 200–400 mg 3-4 times/day (doses more than 1000 mg/day are not well tested in clinical trials). Although NSAIDs provide effective symptomatic treatment for OA, the use of traditional NSAIDs is limited due to their potential life threatening gastrointestinal tract (GIT) adverse effects especially in elderly patients [8]. COX-2 specific inhibitors (Coxibs) have fewer GIT adverse effects but have higher cardiovascular toxicity [9]. 

Eperisone hydrochloride is a beta-amino propiophenone derivative established as an effective centrally acting muscle relaxant. It relieves muscle ischemia and reduces pain [10]. The recommended oral dose for adults is 150 mg per day administered daily in three divided doses of 50 mg each after meals and it is well tolerated. The main adverse effects are gastrointestinal disturbances, light headedness, and sleepiness [11]. 

Joint pain of OA knee is localized deep ache that worsens on activity. Apart from synovitis, stretching of nerve endings in the periosteum, microfractures in subchondral bone, joint instability leading to stretching of joint capsule, and muscle spasm may also be source of pain [12]. Adequate studies have not been conducted to examine the role of muscle relaxation to afford relief of pain in OA knee. However, they are commonly used in practice. OA is a disease of the elderly, and use of muscle relaxants can cause light headedness and sedation which can lead to falls. It is also not clear whether the addition of muscle relaxants to analgesics can improve upon the efficacy of NSAID alone. Therefore, the aim of present study was to find out if the addition of muscle relaxant to the NSAID adds to the efficacy and to compare safety of using combination over NSAID alone in patients of OA knee.

## 2. Patients and Methods

We conducted an 8-week, randomized, open label, parallel group, comparative study in patients of OA knee joint, who reported at outpatient Department of Orthopaedics, Government Medical College and Rajindra Hospital, Patiala. The study was approved by Research and Ethics Committee of the institution. The study is duly registered with the Clinical Trial Registry of India vide registration number CTRI/2013/03/003442. A total of 60 patients who met all the inclusion criteria and had none of the exclusion criteria were enrolled after obtaining written informed consent (see Table 1). 

Randomization was done by using computer-generated random list in 1 : 1 ratio. Thirty patients received etodolac 600 mg once daily for 8 weeks and 30 patients received eperisone 50 mg thrice daily in addition to etodolac 600 mg once daily for 8 weeks. Patients in the etodolac group didn't receive any placebo medication. Treatment allocation was concealed; sealed envelopes were opened at the time of allocation. No other drug commonly used for treatment of osteoarthritis was allowed during the study. Patients already taking other analgesic drugs were included in the study after washout period of 1 week. Data was collected on patient's demographic characteristics, functional status involving different parameters like pain intensity, joint tenderness, swelling, erythema, pain on movement, functional capacity, and overall assessment of arthritic condition.

### 2.1. Efficacy and Tolerability Assessment

Primary efficacy endpoint was difference in VAS score at the end of 8 weeks between the two groups. The classic version of the VAS was administered: 10-centimeter line, horizontal. VAS on present pain ranged from “no pain” to “the worst pain possible” and VAS on pain relief ranged from “no pain relief” to “the maximum pain relief.” Scores ranged from 0 to 10 [13].

Secondary efficacy endpoints were difference of scores in joint tenderness, swelling, erythema, pain on movement, functional capacity, and overall assessment between the two groups at the end of 8 weeks. Patients were assessed for functional status at visit one, that is, 0 week (baseline visit), then visit 2 (after 4 weeks), and visit 3 (after 8 weeks) by using Likert version of 5-point scale where “0” represents “none,” “1” is “mild,” “2” is “moderate,” 3 is “severe,” “4” is “extreme” [14]. Investigator assessed the patient's overall arthritic condition using a 5 point scale where: 1 is very good, 2 is good, 3 is fair, 4 is poor, and 5 is very poor [15].

Primary safety endpoint was sedation. Complete physical examination and laboratory evaluation, including blood chemistry profile and urinalysis was done at the baseline and final visits. All the adverse events reported by all patients were noted at follow up visits.

### 2.2. Statistical Analysis

Sample size was calculated considering the pain intensity on VAS as the primary efficacy parameter. To detect a difference of 1 in VAS score between the two groups, assuming a standard deviation of 1 and *α* value of 0.05 at 90% power, the sample size for each group was calculated to be 22. Assuming dropouts and withdrawals (at about 15%–20%), it was decided to enroll 30 patients in each group.

Data are presented as mean ± S.D. The primary efficacy endpoint was analysed using unpaired students *t*-test. All other efficacy endpoints were analysed using unpaired *t*-test for difference between the groups and paired *t*-test for within group analysis. Chi square test was used for categorical variables.

## 3. Results

The demographic and baseline clinical characteristics of study patients were similar between the two groups (Table 2).

**Figure 1:**
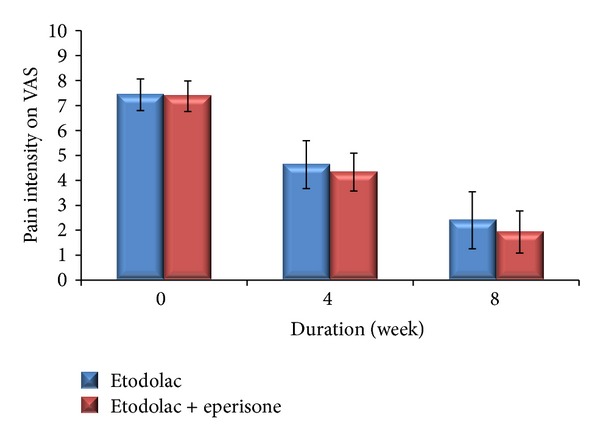
Comparison of pain intensity on VAS in both the groups throughout the study period of 8 weeks.

Both the treatment groups showed a significant reduction in VAS score for spontaneous pain from baseline across time at the end of 8 weeks (*P* < 0.001). In the etodolac group pain score on VAS decreased from 7.43 ± 0.63 to 2.40 ± 1.14 and in the combination group VAS score decreased from 7.37 ± 0.61 to 1.93 ± 0.84 (Figure 1).

In all the secondary efficacy parameters like pain on movement, joint tenderness, swelling, joint erythema, functional capacity, and overall assessment scores, significant improvement was seen within the groups as compared to the baseline. However, there was no significant difference between the groups (Table 3).

Both treatments were well tolerated; adverse events reported were few and mild in nature. No abnormalities were found in the lab investigations. Sedation was seen in 3 patients on the combination therapy, whereas it was not reported by any patient in etodolac group. There was no significant difference in the incidence of adverse events in the two treatment groups (Table 4).

## 4. Discussion

Osteoarthritis is becoming increasingly prevalent worldwide because of the combination of an ageing population and the epidemic of obesity. Although NSAIDs form a major part of the pharmacological therapy of osteoarthritis knee, we could not find any published literature about the role of muscle relaxants in such patients. Therefore, the present study was conducted to explore the potential use of muscle relaxant as adjuvant analgesic in patients of OA knee. It was concluded from the results of our study that both etodolac alone and etodolac in combination with eperisone hcl were effective in OA knee, with each treatment group showing significant improvement in all efficacy parameters throughout the study. Both the drugs were well tolerated and systemically safe as none of the study participants discontinued drug treatment during the entire study period. Moreover, eight weeks is a sufficiently long duration to establish effectiveness of a treatment, and since multiple parameters were tested but none of them showed a significant difference between the groups, so it was also concluded that additional use of muscle relaxant does not provide superior pain relief over NSAID use alone in patients of OA knee, and use of combination is not recommended. But at the same time, we can't rule out the possibility that the therapeutic effect of etodolac may be too good to overshadow any advantage of eperisone so that the advantage of the latter is masked. Our study results are in conformity with results of a double-blind study of benorylate and chlormezanone in musculoskeletal disease wherein the authors concluded that there is no advantage in adding chlormezanone in patients of OA of hip or knee, but there was a significant improvement in pain relief in patients with neck pain [16]. Our study had limitation of being an open label design. Secondly, since OA significantly affects the patient's daily activities, so we should have used more disease specific tools for assessment of patient's functional status that are based on objective criteria. Probably that would have added another important dimension of patient reported outcome to our study. Based on the results of our study, routine use of muscle relaxants cannot be recommended along with NSAIDs in patients of osteoarthritis knee.

## Figures and Tables

**Table 1:** 

Inclusion criteria	Exclusion criteria
Age 35–65 years Both genders (M/F) confirmed diagnosis of OA knee on X-ray	Patients with other forms of inflammatory arthritis Patients with active peptic ulcer, significant renal, hepatic, hematological, or cardiovascular disease H/O hypersensitivity to NSAIDs H/O of skin disorders aggravated by drugs Patients of myasthenia gravis Pregnant women and nursing mothers

**Table 2:** Demographic and baseline clinical characteristics of 60 patients.

Characteristics	Etodolac group (*n* = 30)	Etodolac + eperisone group (*n* = 30)	*P* value (*NS)
Gender			
Male	8	11	0.198*
Female	22	19	
Age in years	51.33 ± 8.14	51.87 ± 6.88	0.594*
Weight in kg	63.07 ± 5.84	61.93 ± 5.44	0.125*
Disease duration (years)	2.19 ± 1.15	1.84 ± 1.23	0.144*
Pain (VAS) score	7.43 ± 0.63	7.37 ± 0.61	0.440*
Joint tenderness score	2.63 ± 0.49	2.50 ± 0.51	0.163*
Swelling score	1.87 ± 0.68	1.90 ± 0.84	0.844*
Erythema score	1.80 ± 0.66	1.87 ± 0.82	0.453*
Pain on movement score	2.43 ± 0.57	2.37 ± 0.56	0.401*
Functional capacity score	2.57 ± 0.57	2.43 ± 0.50	0.163*
Overall arthritic condition score	3.53 ± 0.57	3.47 ± 0.51	0.384*

Data are in mean ± S.D.

**Table 3:** Secondary efficacy variable assessment across time in both the groups.

Efficacy variables (Mean ± SD)	Visits	Etodolac group (*n* = 30)	Etodolac + eperisone group (*n* = 30)	Difference between the groups (*P* value)
Joint tenderness score	Baseline At 8 weeks	2.63 ± 0.49 1.43 ± 0.80	2.50 ± 0.51 1.07 ± 0.65	0.086
Swelling score	Baseline At 8 weeks	1.87 ± 0.68 1.07 ± 0.25	1.90 ± 0.84 1.00 ± 0.00	0.107
Erythema score	Baseline At 8 weeks	1.80 ± 0.66 1.07 ± 0.25	1.87 ± 0.82 1.03 ± 0.18	0.232
Pain on movement score	Baseline At 8 weeks	2.43 ± 0.57 1.23 ± 0.43	2.37 ± 0.56 1.03 ± 0.48	0.097
Functional capacity score	Baseline At 8 weeks	2.57 ± 0.57 1.27 ± 0.52	2.43 ± 0.50 1.10 ± 0.31	0.107
Overall arthritic condition score	Baseline At 8 weeks	3.53 ± 0.57 1.70 ± 0.70	3.47 ± 0.51 1.50 ± 0.57	0.135

**Table 4:** Comparison of reported adverse events between the groups.

Adverse effects	Etodolac group	Etodolac + eperisone group
Constipation	1	1
Diarrhoea	1	3
Epigastric pain	2	5
Heart burn	3	0
Indigestion	2	6
Nausea	1	0
Headache	0	2
Tiredness	0	1
Sedation	0	3

Total adverse events	10	21
